# Tuning of composition and morphology of LiFePO_4_ cathode for applications in all solid-state lithium metal batteries

**DOI:** 10.1038/s41598-022-09244-3

**Published:** 2022-03-31

**Authors:** Harimohan Erabhoina, Mukundan Thelakkat

**Affiliations:** 1 Applied Functional Polymers, University of Bayreuth, Universitätsstraße.30, 95447 Bayreuth, Germany; 2 Bavarian Polymer Institute, University of Bayreuth, Universitätsstraße.30, 95447 Bayreuth, Germany; 3 Bavarian Centre for Battery Technology (BayBatt), University of Bayreuth, Universitätsstraße.30, 95447 Bayreuth, Germany

**Keywords:** Energy science and technology, Energy storage, Batteries

## Abstract

All solid-state rechargeable lithium metal batteries (SS-LMBs) are gaining more and more importance because of their higher safety and higher energy densities in comparison to their liquid-based counterparts. In spite of this potential, their low discharge capacities and poor rate performances limit them to be used as state-of-the-art SS-LMBs. This arise due to the low intrinsic ionic and electronic transport pathways within the solid components in the cathode during the fast charge/discharge processes. Therefore, it is necessary to have a cathode with good electron conducting channels to increase the active material utilization without blocking the movement of lithium ions. Since SS-LMBs require a different morphology and composition of the cathode, we selected LiFePO_4_ (LFP) as a prototype and, we have systematically studied the influence of the cathode composition by varying the contents of active material LFP, conductive additives (super C65 conductive carbon black and conductive graphite), ion conducting components (PEO and LiTFSI) in order to elucidate the best ion as well as electron conduction morphology in the cathode. In addition, a comparative study on different cathode slurry preparation methods was made, wherein ball milling was found to reduce the particle size and increase the homogeneity of LFP which further aids fast Li ion transport throughout the electrode. The SEM analysis of the resulting calendered electrode shows the formation of non-porous and crack-free structures with the presence of conductive graphite throughout the electrode. As a result, the optimum LFP cathode composition with solid polymer nanocomposite electrolyte (SPNE) delivered higher initial discharge capacities of 114 mAh g^-1^ at 0.2C rate at 30 °C and 141 mAh g^-1^ at 1C rate at 70 °C. When the current rate was increased to 2C, the electrode still delivered high discharge capacity of 82 mAh g^-1^ even after 500 cycle, which indicates that the optimum cathode formulation is one of the important parameters in building high rate and long cycle performing SS-LMBs.

## Introduction

Although lithium-ion batteries (LIBs) have achieved impressive success in the past years, the energy density that is gradually approaching the theoretical limit in liquid electrolyte-based systems still cannot meet the actual requirements of electrical energy vehicles. Therefore, there is renewed interest in using pure lithium metal as anode, which necessitates the adaptation of electrolytes to mechanically stable solid-state electrolytes as well as cathodes towards all solid-state lithium metal batteries (SS-LMBs)^1^. This is based on the fact that the lithium anode in SS-LMBs can reach ultra-high theoretical specific capacity (3860 mAh g^-1^), low density (0.53 g cm^-3^) and lowest electrochemical potential (−3.04 V vs SHE). In addition to high energy density, the SS-LMBs can also assure high safety when compared to the conventional LIBs having flammable organic solvents^1^. In spite of this huge prospect, a rapid commercialization of SS-LMBs is still hindered due to issues such as their low ionic conductivity (presence of all solid components in the cell), low discharge capacity at higher current rates and poor cycle life^1,2^. Although a tremendous amount of work has been carried out on solid state electrolytes to improve their ionic conductivities, and chemical, mechanical and electrochemical stabilities within the operating potential window of the electrode^1,3^, less concentration has been paid on the engineering aspects of the cathode for SS-LMBs.

Among all the cathode materials of lithium-ion battery (LIB) family, LiFePO_4_ (LFP) is one of the potential candidates from the application point of view due to its appreciably good theoretical discharge capacity of 170 mAh/g, flat operating potential (3.4 V vs Li^+^/Li), excellent reversibility, low cost, environmental benignity and high thermal and chemical stabilities^4^. Therefore, LFP is used as standard cathode material in the present study. Typically, the LFP content in the cathode used for liquid-based systems are in the range of 80–85 wt%^5–7^. The high active material loading ends up with high porous structures in the electrode which leads to ohmic contact resistance and poor rate performance. Therefore, an optimum porosity was maintained in the electrode by calendaring process (densification of electrode) in order to have better contact between the particles as well as with minimum porosity for liquid electrolyte accessibility for Li ion movement during the redox process^7^. Though, the LFP cathode composition for the liquid-based systems is well studied and standardized in order to attain better electrochemical performance with good cycle stability^8,9^, the LFP cathode composition and morphology need to be varied to obtain the required compact (non-porous) morphology for SS-LMB. For example, the pores in the cathode have a negative impact on the electrochemical performance of SS-LMB, because the porous structures do not get filled with solid electrolytes, which in turn impede the lithium ion and electron percolation pathways^10^.

**Figure 1:**
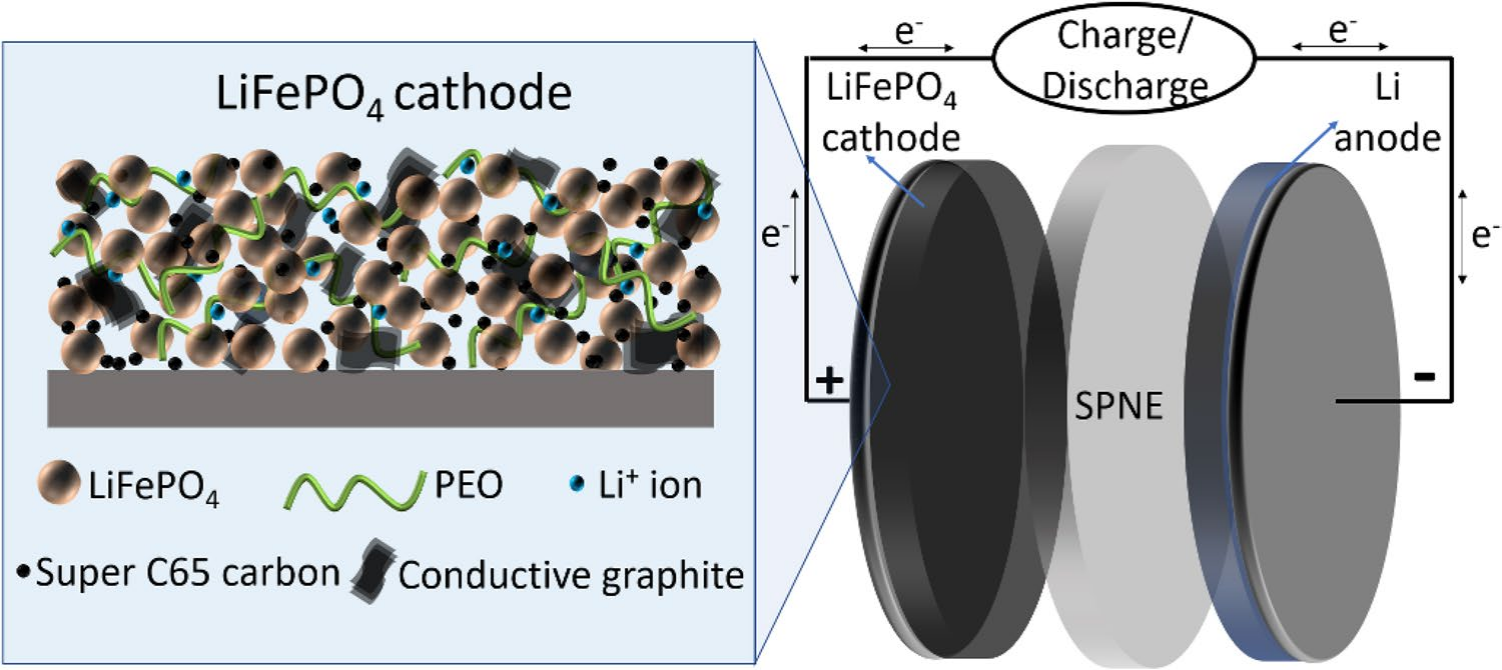
Schematic illustration of uniformly distributed cathode components (left) and all solid-state lithium metal battery SS-LMB (right).

In LIBs, the standard cathode composition consists of active material (for example; LFP), carbon black and polyvinylidene difluoride (PVDF) binder^6^. However, the same composition cannot be suitable for SS-LMBs because the cathode needs both ion and electronic conducting mediums in it (Fig. 1). In general, the cathodes used for PEO-based systems that are reported in the literature have different amounts of active material, ion and electron conductive additives as electrode composition^11–13^. For example, Wan et al.^11^ reduced the LFP content to 60 wt% and obtained a stable discharge capacity of ~ 100 mAh g^-1^ even after 300 cycles at 0.5C at 60 °C. This is supported by later studies, which also used 60–70 wt% of LFP for a stable electrochemical performance^13–15^. Though, the cells fabricated by these electrodes exhibited good electrochemical performance, the reason for selecting a particular composition to obtain optimum amount of each component in the cathode as well as its consequence on morphology is not reported, which motivated us to study the influence of composition and morphology on the electrochemical performance of the LFP cathode during higher C rates. It is also reported in the literature that the addition of PEO in the cathode has multifunctional effects such as it enhances the ionic conductivity via hopping mechanism^1^, acts as binder for all the components in the electrode and also helps in integrating both the cathode and electrolyte layers to form a stable structure^16^. However, the electronic conductivity is mainly dependent on the type of conductive additives used, which plays an important role in enhancing the overall electrochemical performance such as rate capability and cycle stability of the cathode material. Recently, Ann et al.^17^, has studied the effect of binary conductive additives such as vapor grown carbon fibers (VGCF) and Super P carbon on the electrochemical performance of layered cathode (LiNi_0.6_Co_0.2_Mn_0.2_O_2_) material for SS-LMBs. They concluded that the presence of fibrous VGCF with intact nanostructured carbon particles and active material in the cathode increases the rate performance of the cell due to continuous electron conduction networks. Therefore, it is desirable to choose the conductive additive, which can improve the electrical conductivity at high current rates and should be of low cost from the commercial point of view.

In addition to electrode composition, it is challenging to prepare the cathode electrode for SS-LMBs with non-porous structure for better ion conduction during the fast reaction kinetics and also the homogenous distribution of active material and conductive particles within the binder matrix for good electron transport pathways^18^. In general, liquid-based slurry casting process is the most commonly used method reported in the literature for the fabrication of cathode in SS-LMBs^14,15^. As a result of this, the mechanical contact among the active material, conductive additive and the binder increases, which leads to enhanced electrochemical performance. Furthermore, it is also reported that the particle size of the active material also plays an important role in improving the electrical conductivity and overall electrochemical performance in SS-LMBs^19,20^, paving the way for slurry preparation processes. For instance, Strauss et al.^20^ studied the influence of particle size on the electrochemical behaviour of layered cathode ranging from 4.8 to 15.6 μm. The cell with cathode material of small particle size delivered high discharge capacity of 162 mAh g^-1^ in comparison to the cathode with big particle size (84 mAh g^-1^) and they have concluded that the smaller particles provide better electron percolation pathways within the cathode during the redox process. Another report states that the large active material particle size in the electrode require high content of conductive additive in order to provide continuous electron percolation pathways^21^. Hence considering all these aspects, it is necessary to have a comprehensive understanding of how the cathode components, slurry preparation method and calendaring process influences the quality of the electrode in building high performing batteries in order to meet the requirements of advanced electronics as well as EV applications. Such a systematic and comprehensive study using LFP as a prototype cathode material brings very useful information for the scientific community to create new compositions as well as new systems with any novel cathode material for SS-LMBs.

Herein, we studied the influence of lithium salt concentration, conductive additives, morphology and electrode composition on the electrochemical performance of LFP cathode (as a prototype material) for all SS-LMBs. As electrolyte, we selected a solid polymer nanocomposite electrolyte (SPNE), PEO/LiTFSI (EO/Li = 20) containing 10 wt% of TiO_2_ nanoparticles. Initially, morphological studies were made on electrode to study how calendaring process helps in densifying the electrode to form non-porous structure and its influence on rate performance in a battery. A comparative study on ball milling vs conventional magnetic stirring based slurry preparation processes give an insight on the effect of particle size and their impact on the electrochemical performance of LFP cathode. Furthermore, a systematic study on cathode composition by varying EO/Li ratios of PEO/LiTFSI, super C65 conductive carbon black vs conductive graphite and active material provide better understanding about the electrochemical behaviour of individual components and the corresponding content in the cathode. As a result, the cathode composition of LFP-5 (with an electrode composition of LFP:CB:CG:PEO:LITFSI = 63:4.9:2.1:19.4:10.6, where CB and CG stand for super C65 conductive carbon black and conductive graphite, respectively) cells delivered the highest discharge capacities at 30 °C and 70 °C with good stability upto 50 cycles. Finally, we also studied how the thickness of SPNE influences the discharge capacity of LFP-5 cathode at high current rates.

## Results and discussion

In order to understand the electrochemical behaviour of cathode, the other components in a battery such as electrolyte (SPNE) and lithium metal anode are used as a reference standard throughout the measurements. The EO/Li ratio of PEO/LiTFSI is maintained as 20:1 according to the literature report^22^, due to its good mechanical stability, high lithium ion transport number and high ionic conductivity. To start with, the cathode composition was adapted from the reported literature, wherein Judez et al.^22^ have used 63 wt% of LFP as active material. Thus, the first LFP cathode (LFP-1) has a wt% composition of LFP:CB:PEO:LiTFSI = 63:7:22.7:7.3, where CB stands for super C65 conductive carbon black (see Table 1).

**Table 1:** Influence of EO:Li ratio in LFP cathode on electrochemical performance.

Sample	Cathode composition		Method	EO:Li ratio	Capacity (mAh g^-1^)
LFP-1	LFP:CB:PEO:LiTFSI	63:7:22.77.3	Magnetic stirring	20:1	82 (1C)
LFP-2	LFP:CB:PEO:LiTFSI	63:7:21.4:8.6	Magnetic stirring	16:1	99 (1C)
LFP-3	LFP:CB:PEO:LiTFSI	63:7:19.4:10.6	Magnetic stirring	12:1	107 (1C)

The batteries were tested at different C rates (ranging from 0.1C to 1C) at 70 °C for 30 cycles using Li/SPNE/LFP configuration.

### Importance of calendaring process on morphology of LFP cathode

**Figure 2:**
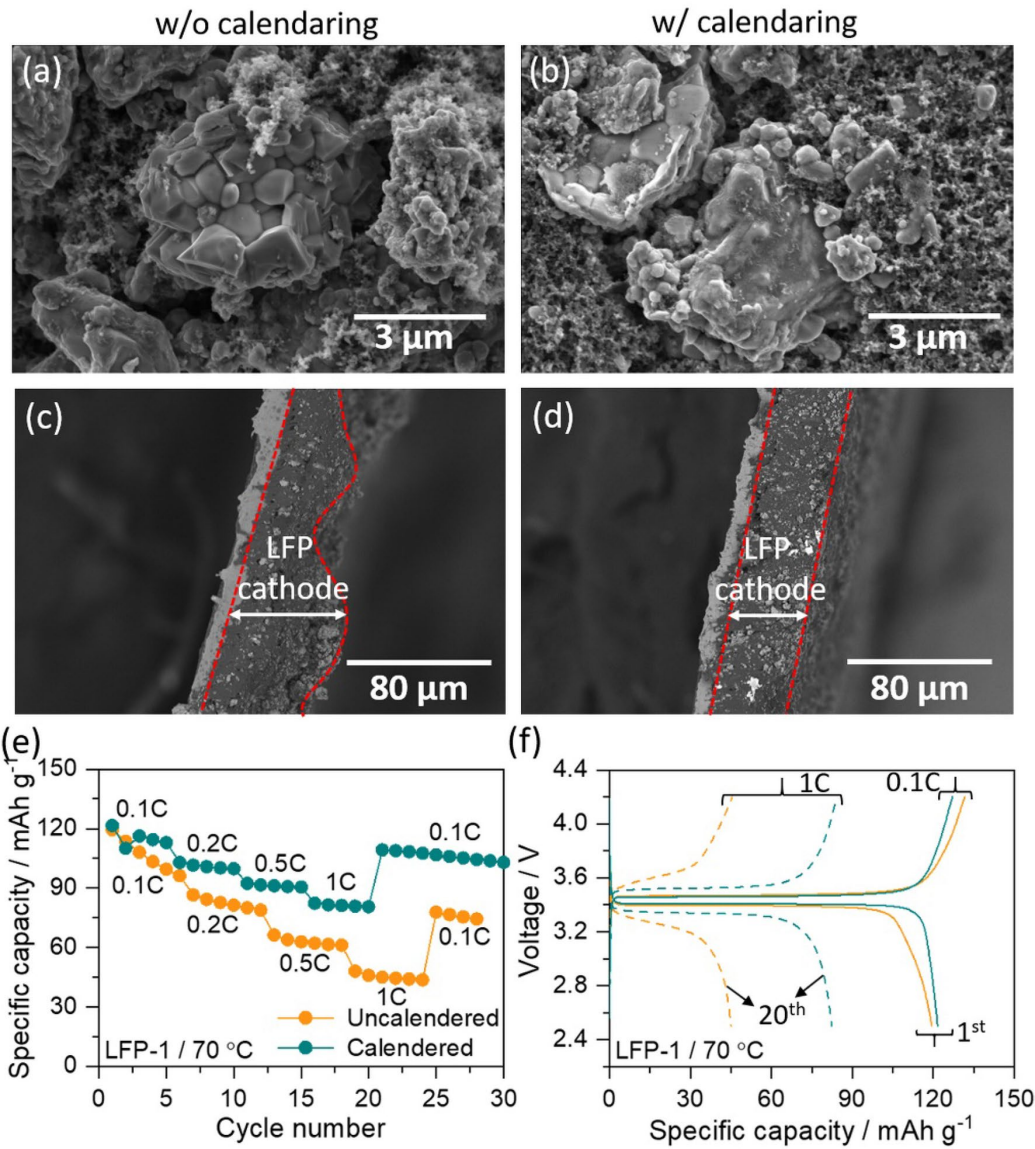
(**a**) and (**b**) are the SEM images, (**c**) and (**d**) are the cross-sectional SEM images of LFP-1 cathode before and after calendaring process, (**e**) comparative rate capability curve and (**f**) the corresponding charge/discharge curves of Li/SPNE/LFP cell using LFP-1 measured at 70 °C at different current rates, from 0.1C to 1C.

The LFP-1 slurry prepared by magnetic stirring was coated on to carbon-coated aluminium foils, dried and subjected to calendaring process. These LFP-1 cathode layers with and without calendaring was subjected to SEM analysis in order to understand the morphology, porosity and distribution of active particles in the cathodes. Figure 2a, b are the SEM images of LFP-1 before and after calendaring process. Both uncalendered and calendered electrodes show large agglomerated LFP particles with non-uniform distribution. However, the uncalendared electrode displays high porosity and cracks in the electrode, which were reduced after calendaring process (Figure S1). The dense structure provides all the components in the electrode to be intact and that helps in better ion and electron conduction pathways during the redox process. Furthermore, the cross-sectional SEM images of uncalendered LFP-1 (Fig. 2c) shows an uneven surface, whereas the calendered electrodes (Fig. 2d) shows smooth surface. The flat and smooth surface of the electrode is expected to reduce the interfacial resistance between the cathode and electrolyte layers during cycling process. The influence of calendaring the electrode was clearly observed during the rate capability measurements as shown in the Figs. 1f, 2e. At 0.1C, both the cells delivered comparable initial discharge capacity (uncalendered LFP-1 = 119 mAh g^-1^; calendered LFP-1 = 121 mAh g^-1^) at 70 °C. However, a huge difference is observed with increase in the current rate to 1C. The calendered LFP-1 cell delivered 82 mAh g^-1^ in comparison to the uncalendared electrode (47 mAh g^-1^). Moreover, the uncalendared electrode displays large overpotential (Fig. 2f) indicating that the porous structures in the cathode reduces the lithium ion movement during fast reaction kinetics. Thus, a densification and decrease in porosity is the first requirement for SS-LMBs. Henceforth, all the cathodes were calendered before their electrochemical measurements.

### Variation of Li-salt content in cathode

**Figure 3:**
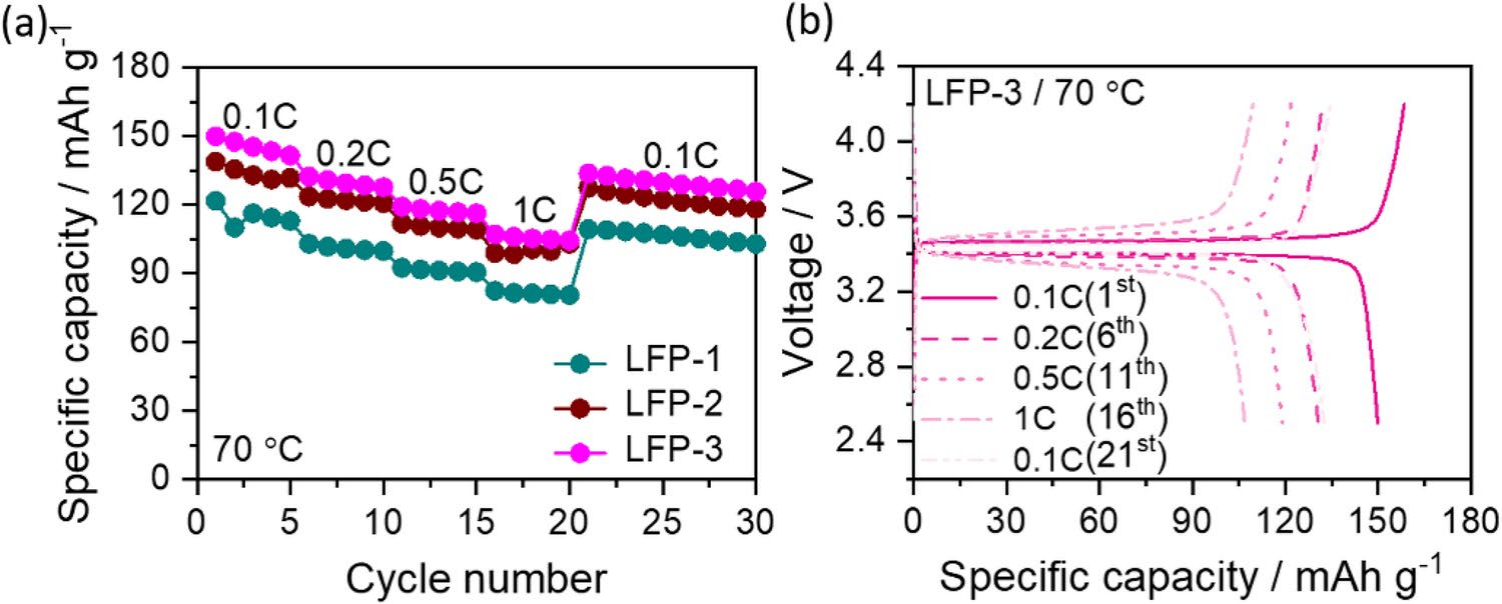
(**a**) Comparative rate capability curves of Li/SPNE/LFP cells having LFP-1 to LFP-3 cathodes and (**b**) the corresponding charge/discharge curves for LFP-3 cathode measured at 70 °C at different current rates.

The lithium salt content in the cathode can influence the discharge capacity and also the cyclic stability during repeated charge/discharge processes. To verify and understand the necessity and influence of Li-salt content in cathode, the LFP content was kept constant (63 wt%) and the three different weight ratios of PEO:LiTFSI were selected during the preparation of cathode slurry which corresponds to the EO/Li ratios of 20:1 (LFP-1), 16:1 (LFP-2) and 12:1 (LFP-3), respectively (Table 1). It is to be noted that the PEO with different salt contents used in the cathodes show semicrystalline behavior with decreasing crystallinity on increasing salt content (see figure S2 and table S1). As given in table S1, the melt enthalpy (ΔH_m_), which is a measure of crystallinity reduces from 57 to 4 J.g ^−1^ for samples with EO/Li ratios from 20:1 to 12:1 respectively. For the same range of salt content variation, the T_g_ decreases from −33 to −40 °C respectively. This is in accordance with an earlier report of Polu et al.^23^, who also observed that an increase in the lithium salt content in the PEO polymer results in reduced degree of crystallinity and a decrease in T_g_ due to the formation of transient bridges between the ether oxygen groups and the salt. This also helps in improving the ionic conductivity at low temperature regions. All the slurries were prepared by magnetic stirring process. Figure 3a shows the comparative rate capability curves of LFP-1 to LFP-3 electrodes measured at different current rates ranging from 0.1C to 1C at 70 °C. It is interesting to note that the capacity of the cell increases with increasing lithium salt content in the electrode and the resulting cells with LFP-1, LFP-2 and LFP-3 electrodes delivered high initial discharge capacities of 121 mAh g^-1^, 138 mAh g^-1^, and 149 mAh g^-1^, respectively. Even at high current rate of 1C the electrodes delivered an appreciated discharge capacity of 82 mAh g^-1^, 99 mAh g^-1^ and 107 mAh g^-1^ and retained the capacities of 103 mAh g^-1^, 118 mAh g^-1^, and 126 mAh g^-1^, respectively, when the current rate is reversed back to 0.1C. In the present study, we limited the EO/Li ratio to 12:1, because it is reported that the increased EO/Li ratio beyond eutectic (9:1) leads to phase segregation which may leads to poor cycle performance^24^. Thus, the EO/Li ratio of 12:1 is selected as optimum content during the preparation of cathodes. Figure 3b shows a typical charge/discharge profile of the corresponding LFP-3 electrode. The charge/discharge profile show a clear plateau around 3.45 V which represents the typical redox behavior of LFP cathode^4^.

The high discharge capacity of LFP-3 is mainly due to sufficient lithium ion concentration, non-porous and crack free structure of the electrode which increases both the ion and electron conduction pathways at high current rate (1C). As already mentioned in the introduction that the large particle size of active material has huge negative impact on the electrochemical properties due to limited lithium ion diffusion into the solid-state agglomerated particles. A similar behavior is also observed in the present study, with increasing the current rate the discharge capacity decreases owing to nonhomogeneous distribution of agglomerated LFP particles in the cathode (supported by the SEM analysis). Therefore, the slurry preparation process may play an important role to decrease the LFP particle size and improve the homogeneity to achieve an enhanced electrochemical performance of the cell.

### Ball milling vs. magnetic stirring of slurry

Now the slurry preparation process was varied and compared to study its influence using the selected EO:Li ratio of 12:1 on Li/SPNE/LFP cells. To understand the influence of LFP particle size on the electrochemical performance, we applied ball mill method on our standardized slurry preparation process. For this, two samples were comparatively studied, namely LFP-3 and LFP-4, where LFP-3 was obtained after magnetic stirring, whereas LFP-4 by a ball milling process. The exact compositions are kept the same and are described in Table 2. It is reported in the literature (for liquid electrolyte batteries) that the ball milling method helps in decreasing the particle size and also increase the homogeneity of active particles throughout the electrode^25,26^. For this study, the electrode composition of LFP-3 is used as a standard and the resulting mixture was ball-milled for 2 h (Table 2).

**Table 2:** Influence of the slurry preparation method (EO:Li = 12:1) on electrochemical performance.

Sample	Cathode composition		Method	Initial capacity (mAh g^-1^)	Final capacity (mAh g^-1^) at 50th cycle
LFP-3	LFP:CB:PEO:LITFSI	63:7:19.4:10.6	Magnetic stirring	115	77
LFP-4	LFP:CB:PEO:LITFSI	63:7:19.4:10.6	Ball milling	137	109

The batteries were tested at 1C at 70 °C for 50 cycles using Li/SPNE/LFP configuration.

**Figure 4:**
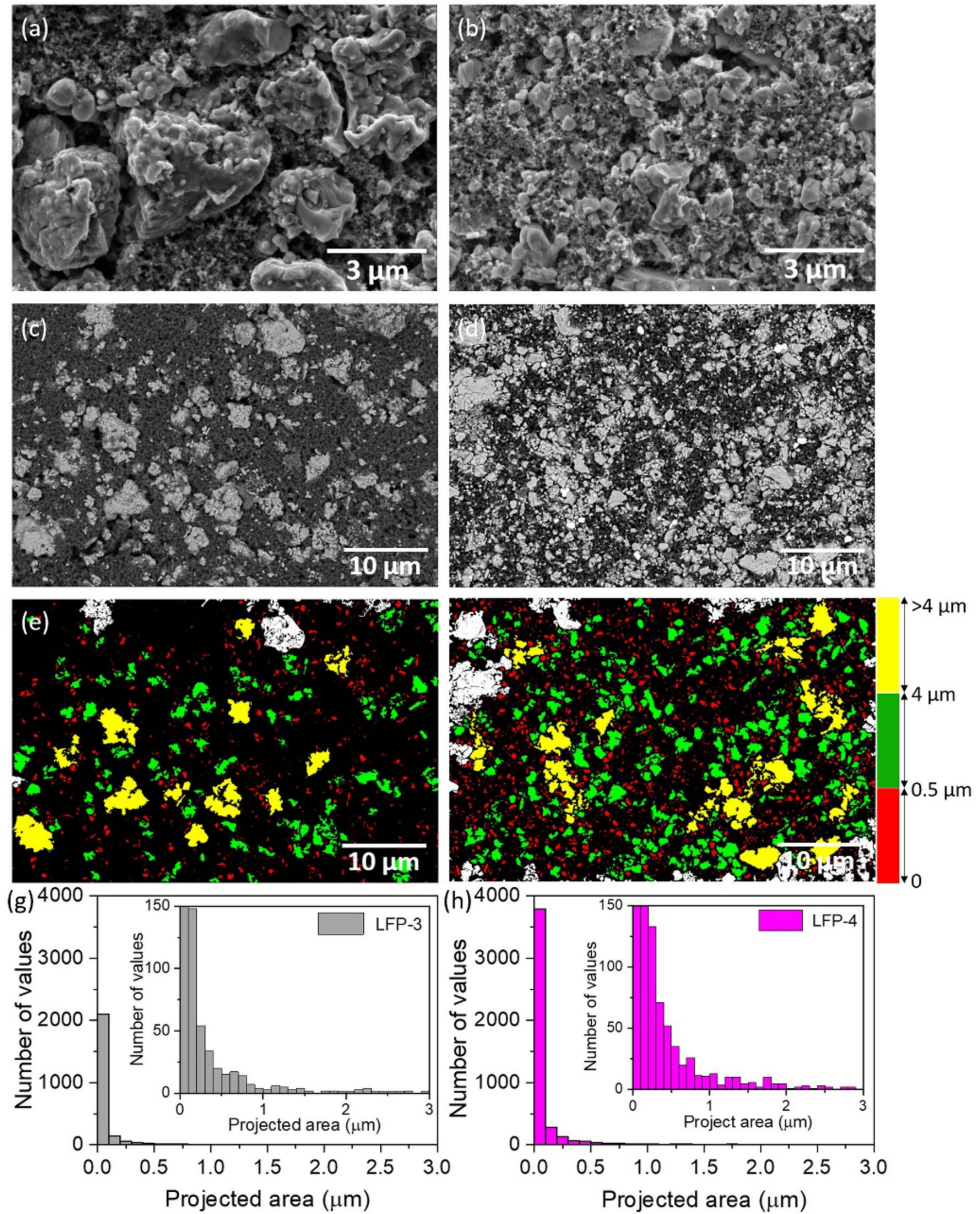
(**a**) and (**b**) are the SEM images of calendered LFP-3 and LFP-4 cathodes prepared by using magnetic stirring and ball milling processes, (**c**) and (**d**) are the SEM back scattered images, (**e**) and (**f**) are the particle size distribution with color codes defining the sizes and (**g**) and (**h**) are the corresponding projected area analysis of LFP-3 and LFP-4 cathodes indicating the number of particles.

**Figure 5:**
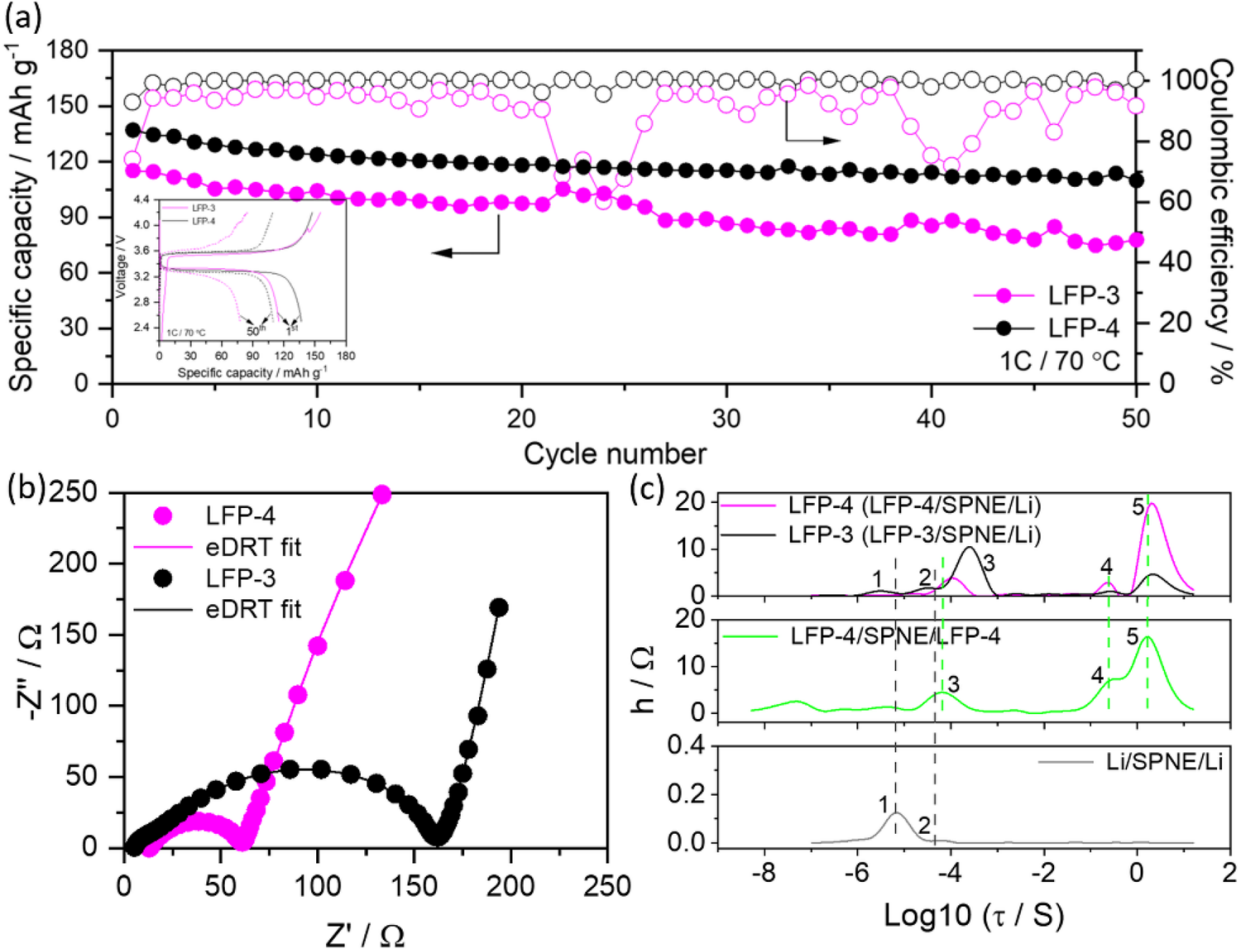
(**a**) Comparative cycle performance curves of Li/SPNE/LFP batteries having LFP-3 and LFP-4 as cathodes measured at 70 °C at 1C rate with inset of their corresponding charge–discharge curves for the 1st and 50th cycles, (**b**) the corresponding Nyquist plots before cycling and (**c**) a comparison of the eDRT time constants curves of the batteries (top) with symmetrical cathode-only cell (middle) and anode-only cells (bottom), all measured at 70 °C.

The detailed method of preparation is given in the experimental section. SEM analysis were carried out after solvent casting and drying the electrodes on carbon-coated aluminium substrates. Figure 4a, b shows a comparative SEM images of calendered LFP-3 and LFP-4 electrodes. It is clearly observed from the SEM images that the particle size of LFP decreases during the ball milling process and also increases the uniformity of all the components in the cathode LFP-4. In order to further support this the samples were analysed at low magnification using back scattered detector to have better understanding about the particle size distribution. The bright areas in the Fig. 4c, d corresponds to the LFP particles which clearly indicates that the particle size of LFP decreased during milling process and distributed homogenously throughout the electrode, whereas, the LFP in magnetic stirred sample shows nonhomogeneous agglomerated particles (Figure S3). Further, the particle size analysis was conducted by MountainsSEM Expert V9 in order to see the qualitative distribution of LFP in the electrodes. The red, green and yellow colours represent the particle size range from ≤ 0.5 μm, 0.5 μm–4 μm, and ≥ 4 μm, respectively, in the samples (Fig. 4e, f). The larger extend of red and green colours (smaller sizes) in LFP-4 sample and the increased number of particles in the projected area as given in Fig. 4h (number of values represents the number of particles in y axis) are obvious in LFP-4 in comparison to LFP-3 (Fig. 4g) indicating a preferential distribution of particle size ranging from nano to micron scale (≤ 4 μm) in LFP-4. This directly influences the electrochemical performance as shown in the Fig. 5a. The Li/SPNE/LFP-4 cell delivered high initial discharge capacity of 137 mAh g^-1^, in comparison to Li/SPNE/LFP-3 cell (115 mAh g^-1^) at 1C rate at 70 °C. The enhanced electrochemical performance of Li/SPNE/LFP-4 cell could be attributed to the smaller LFP particles which increases the Li ion diffusion as well as electron percolation pathways more efficiently at high current rate. The influence of cathode particle size on the electrochemical performance of SS-LMB has been recently studied by Strauss et al.^20^, and reported that the ionic conductivity is not affected by the particle size, but the electronic conductivity is drastically increased by 3 orders of magnitude by decreasing the particle size from 15.6 to 4.8 μm. In the present study, even after 50 cycles the Li/SPNE/LFP-4 cell delivered the high discharge capacity of 109 mAh g^-1^ in comparison to Li/SPNE/LFP-3 cell with a capacity value of 77 mAh g^-1^ after 50 cycles with good coulombic efficiency.

However, the cells show fluctuations during repeated cycling. It is well reported in the literature that the PEO based electrolytes with the combination of cathodes such as LFP or NMC and lithium metal anode show fluctuations during charge/discharge and cycle performance processes^22,27–30^. This behaviour is more clearly explained by Homman et al.^27^, wherein they have fabricated the cells using different cathode materials (LFP, NMC622, LMO and LNMO), lithium metal anode and 100 µm thick PEO/LiTFSI as solid electrolyte. They have observed the formation of soft lithium dendrites onto the surface of lithium metal anode during charge process which penetrates through the solid electrolyte. This leads to micro shorts in the cell with temporary chemical lithiation of cathode along with the parallel delithiation process, which resulted in voltage fluctuation. Another report states that the dendrite formation takes place due to current inhomogeneities in LFP cells because of the semicrystalline PEO solid electrolyte^31^. A similar trend has been observed in the present study where we have used 50 micros thin PEO-based solid electrolyte which may be associated with the soft dendrite formation during charging due to non-homogenous lithium deposition at high C rate (1C) measurements. The electrochemical result infers that the ball milling is one of the efficient methods for the preparation of cathode slurry for SS-LMB, which helps in decreasing the particle size as well as increases the contact area between particles and thereby improving the overall electrochemical performance of the cell even at higher current rates. The improvement in the electrochemical performance of LFP-4 cathode (prepared by ball milling) was further supported by electrochemical impedance studies. Figure 5b, c shows the Nyquist plots and the corresponding eDRT curves of LFP-3 and LFP-4 cathodes measured before cycling at 70 °C. Both the cells show depressed semicircles in the high-mid frequency region, which correspond to charge transfer resistance at the electrode–electrolyte interface and an incline line at low frequency region representing Warburg impedance, which is associated with the diffusion of Li^+^ ion from the electrode to the electrolyte^32^. On comparison, the LFP-4 cell showed lower charge transfer resistance than LFP-3 cells. Furthermore, the Nyquist plots were fitted with eDRT and compared with symmetrical anode-only (Li/SPNE/Li) and cathode-only (LFP-4/SPNE/LFP-4) cells (Fig. 5c). The corresponding Nyquist plots of the symmetrical cells are given in figures S4a and S4b. This comparison helps to understand the detailed processes in the LFP/SPNE/Li cells having different cathodes. Peak 1 and 2 in Fig. 5c (only time constants available in anode-only device) represents the anodic processes, which attribute to double layer effects and the formation of interfacial layer (SEI layer), whereas peak 3 (additional peak in cathode only device) corresponds to charge transfer resistance at the cathode. Similarly peaks 4 and 5 (absent in anode-only cell) can be assigned to the diffusion processes in the Li/SPNE/LFP cell^33,34^. In LFP-4/SPNE/Li cell, the peak 3 position is shifted towards lower time constant for LFP-4 than LFP-3 with decreased intensity indicating fast reaction kinetics in the LFP-4 cathode in comparison to LFP-3. This result infers that the small particle size with homogenous distribution in the cathode helps in reducing the charge transfer resistance by enhancing the ion and electron conduction pathways.

### Nature of conductive additives: super C65 conductive carbon black vs. conductive graphite

Further, we also studied the influence of different electrically conductive additives (super C65 conductive carbon black, CB and conductive graphite, CG) and their weight ratios on the electrochemical performance of LFP cathode for SS-LMBs. It is reported in the literature for liquid based LIB system that the addition of graphite to LFP cathode helps in increasing the density of the electrode in comparison with super P carbon alone^8^. In addition, the large graphite particle size provides better contact between the active LFP particles and increases the electron percolation pathways more efficiently during fast reaction kinetics. Therefore, in the present study we examined the influence of homogenously distributed graphite particles in LFP cathode in the following.

The total amount of super C65 conductive carbon black (CB) used during the preparation of LFP1-4 cathode is 7 wt%. Therefore, the CB content is decreased systematically and the reduced amount is compensated by conductive graphite (CG) in the series, LFP5-7 and fully replaced by CG in LFP-8. The corresponding electrode compositions with different conductive additives are given in the Table 3.

**Table 3:** Variation of conductive additives keeping EO:Li = 12:1.

Sample	Cathode composition		Method	Initial capacity (mAh g^-1^)	Final capacity (mAh g^-1^) at 50th cycle
LFP-4	LFP:CB:PEO:LITFSI	63:7:19.4:10.6	Ball milling	136	109
LFP-5	LFP:CB:CG:PEO:LITFSI	63:4.9:2.1:19.4:10.6	Ball milling	141	128
LFP-6	LFP:CB:CG:PEO:LITFSI	63:3.5:3.5:19.4:10.6	Ball milling	134	120
LFP-7	LFP:CB:CG:PEO:LITFSI	63:2.1:4.9:19.4:10.6	Ball milling	135	106
LFP-8	LFP:CG:PEO:LITFSI	63:7:19.4:10.6	Ball milling	116	76

The batteries were tested at 1C at 70 °C for 50 cycles using Li/SPNE/LFP configuration.

**Figure 6:**
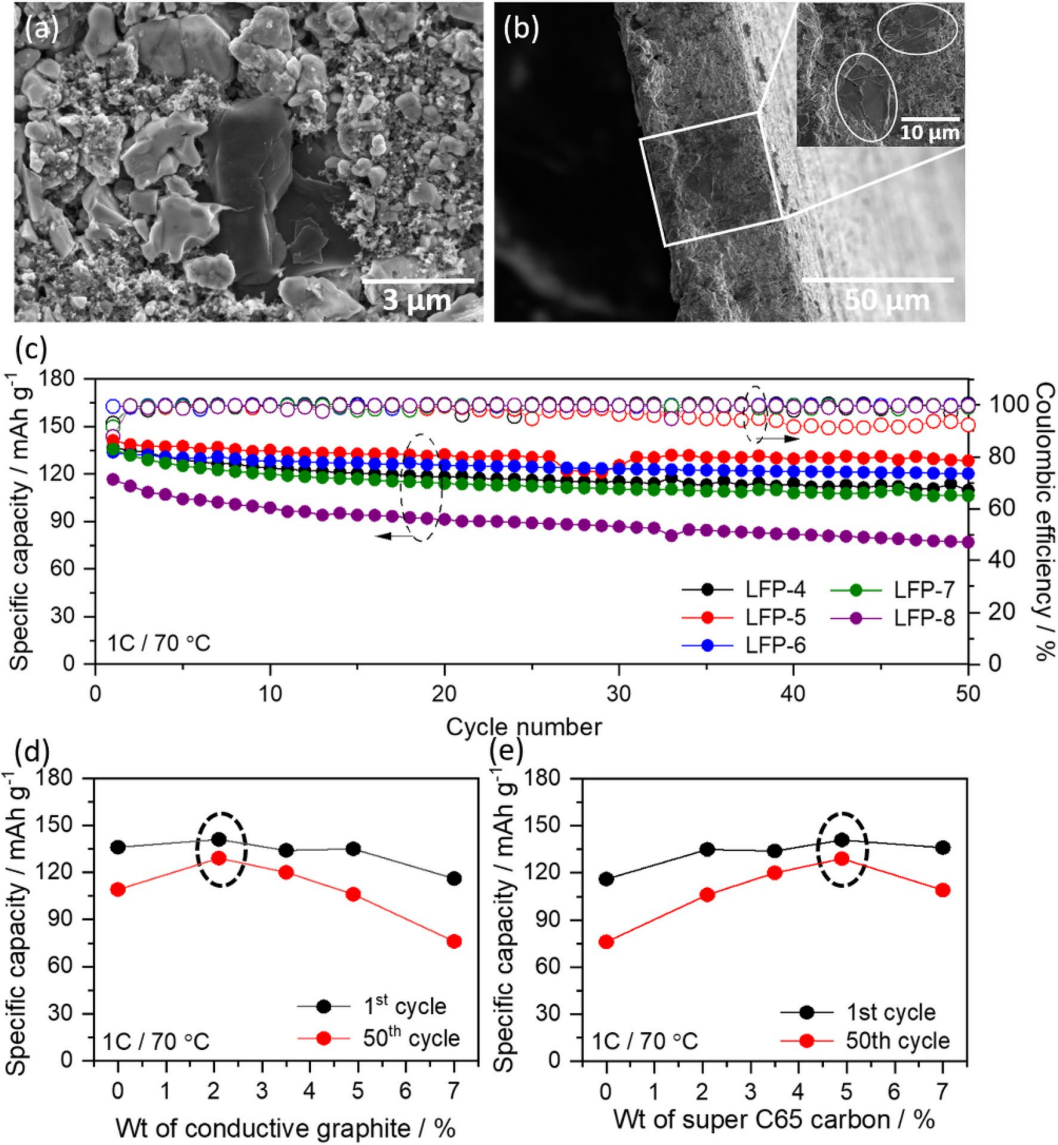
(**a**) and (**b**) are the SEM and cross-sectional image of LFP-5 cathode (**c**) comparative cycle performance of Li/SPNE/LFP cells using LFP-4 to LFP-8 cathodes measured at 1C rate at 70 °C, (**d**) and (**e**) are the comparative graph of specific capacity vs. weight of conductive additives in the electrodes.

After fabricating the electrodes, all the samples were subjected to SEM analysis. Figure 6a shows selected SEM image of LFP-5, and all the other corresponding SEM images of LFP-4, LFP-6 to LFP-7 are given in the supporting information figure S5. It can be clearly observed from the SEM images that the graphite particles (black) are distributed throughout the electrode and are in contact with the surrounding LFP particles (grey). The SEM cross sectional image of LFP-5 shown in Fig. 6b, further supports the uniform distribution of graphite particles in the electrode. As a result of this, the LFP-5 to LFP-7 cathodes also (Fig. 6c) delivered high initial discharge capacities of 141 mAh g^-1^, 134 mAh g^-1^, and 135 mAh g^-1^ which are comparable to that of LFP-4 (136 mAh g^-1^) and retained the capacity values of 128 mAh g^-1^, 120 mAh g^-1^, and 106 mAh g^-1^, respectively at 1C rate at 70 °C with above 95% of coulombic efficiency. However, the LFP-8 delivered comparably the lowest discharge capacities of 76 mAh g^-1^ at 50th cycle. Therefore, the present results infer that the conductive graphite particles which are in close contact with the LFP particles help in increasing the electron transport pathways with the aid of CB particles to the current collector and improves active material utilization. However, the high surface area and high porous structure of CB and high dense structure of CG alone in the electrode (LFP-8) reduces the electron percolation pathways which leads to low specific capacity value. Among them, the LFP-5 cathode with about 2 wt% of CG and 5wt% of CB additives in the electrode (Fig. 6d, e) evolved out to be the suitable weight ratio to achieve better electrochemical performance at high C rates, closely followed by the composition in LFP-6.

### Variation of LFP content in the cathode

**Figure 7:**
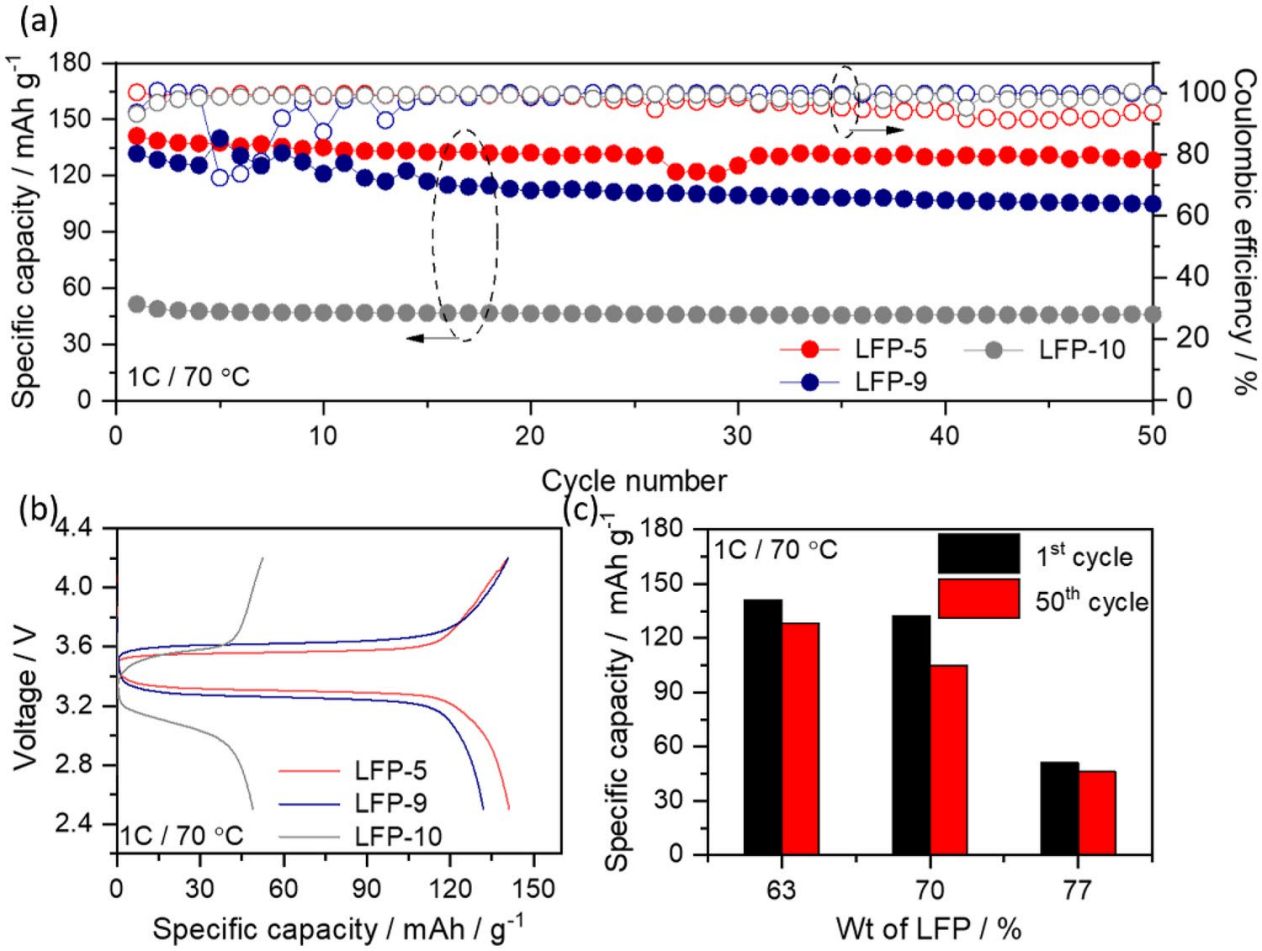
(**a**) Cycle performance curves of Li/SPNE/LFP cells using LFP-5, LFP-9 and LFP-10 cathodes measured at 1C rate at 70 °C, (**b**) the corresponding first charge/discharge curves, and (**c**) comparative specific capacity vs different weight ratios of LFP content in the electrode.

Since all the above studies were carried out with a low amount of 63 wt% LFP, we also addressed the question of how to increase the LFP content and what are the consequences thereof. The active material content in the electrode plays an important role in deciding the specific capacity as well as specific energy of the cell. In this context, the only component which can be reduced is the amount of binder and therefore, we varied the LFP and binder content in the electrode by maintaining carbon and EO:Li (12:1) ratios as constant (as in LFP-5) and the respective electrode compositions are given in Table 4. Thus, LFP content was increased from 63 to 77 wt% from LFP-5 to LFP-10. Initially, the morphological studies were conducted on these electrodes and the corresponding SEM images are given in the supporting information figure S6. The SEM images of LFP-9 and LFP-10 cathodes also shows similar morphological appearance as LFP-5 cathode with the presence of graphite particles throughout the electrode with homogenous distribution. Though the FE-SEM shows similar type of morphological characteristics for all these electrodes, the electrochemical behaviour at 1C rate clearly differentiate the effect of active material content in the electrode. Figure 7a shows the comparative cycle performance curve of LFP-5, LFP-9 and LFP-10 cathodes measured at 1C rate at 70 °C.

**Table 4:** Variation of LFP content keeping EO:Li = 12:1.

Sample	Cathode composition		Method	Initial capacity (mAh g^-1^)	Final capacity (mAh g^-1^) at 50th cycle
LFP-5	LFP:CB:CG:PEO:LITFSI	63:4.9:2.1:19.4:10.6	Ball milling	141	128
LFP-09	LFP:CB:CG:PEO:LITFSI	70:4.9:2.1:14.9:8.1	Ball milling	131	105
LFP-10	LFP:CB:CG:PEO:LITFSI	77:4.9:2.1:10.4:5.6	Ball milling	51	46

The batteries were tested at 1C at 70 °C for 50 cycles using Li/SPNE/LFP configuration.

It is interesting to observe from the Fig. 7a that the LFP-5/SPNE/Li cell showed the highest initial discharge capacity of 141 mAh g^-1^ and retained 128 mAh g^-1^ at 50th cycle, in comparison to, Li/SPNE/LFP-9 cell (131 mAh g^-1^ at 1st cycle, 105 mAh g^-1^ at 50th cycle) and Li/SPNE/LFP-10 cell (51 mAh g^-1^ at 1st cycle, 46 mAh g^-1^ at 50th cycle) at 1C with above 95% of coulombic efficiency. The large differences in electrochemical behaviour can be understood as follows. At high LFP content of 77 wt% in LFP-10, the electron percolation pathways are high enough, but the lithium ion percolation pathways are worse due to low PEO/LiTFSI content, which leads to reduced capacity. On the other hand, the electrode polarization increases with increase in active material as shown in the Fig. 7b. Recently, Kimura et al.^35^ has studied the influence of 55 wt% and 82wt% of active material (LiCoO_2_ cathode) loading on the electrochemical performance of inorganic solid electrolyte based LMB by using computed tomography combined with X-ray absorption near edge structure spectroscopy CT-XANES. This study reveals that the high active material loaded cell shows inhomogeneous reaction distributions due to aggregated active material particles, whereas, this behaviour is not observed in the cell with low active material loading. As a result, the cell with 55wt% active material (see SI Figure S7) delivered high discharge capacity in comparison to the cell with high loading which leads to decreased ionic conductivity during redox process. A similar behaviour has been observed in the present study with increase in active material loading. Therefore, it is concluded from the electrochemical data that the optimum weight ratio of LFP, CB, CG, PEO and LiTFSI are 63, 4.9, 2.1, 19.4 and 10.6, respectively, in order to achieve better electrochemical performance at higher current rates (Fig. 7c). An increase in LFP content is possible only if the Li-ion percolation in cathode is also equally maintained by using better polymer additives other than PEO.

### SPNE thickness variation using LFP-5 cathode

**Figure 8:**
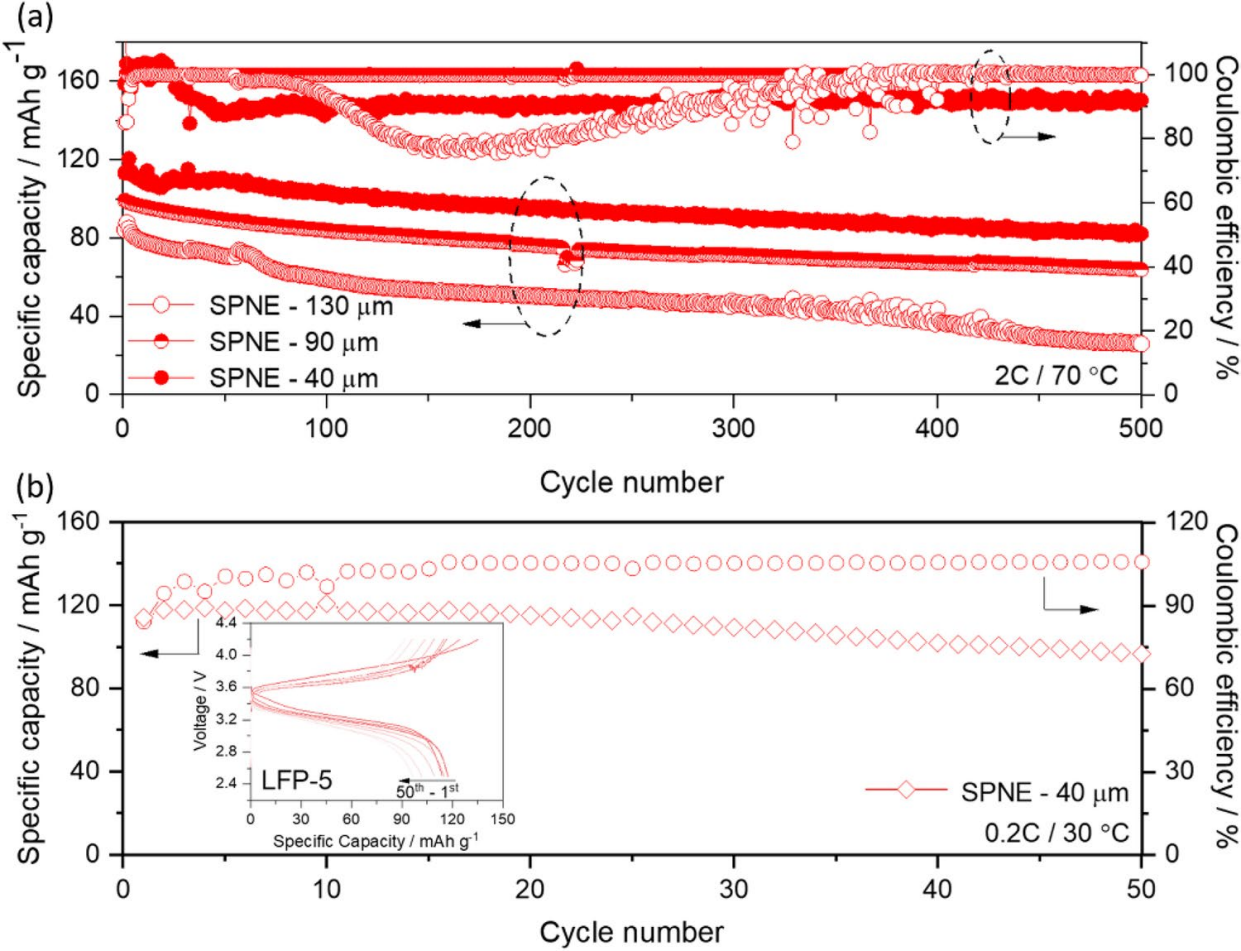
(**a**) Long-term cycle performance curve of Li/SPNE/LFP-5 cell with different thickness of SPNE (40, 90 and 130 µm) measured at 2C rate at 70 °C and (**b**) Cycle performance curve of the same cell measured at 0.2C rate at 30 °C with in-set of its charge/discharge curves for 40 µm thickness of SPNE.

In general, it is reported that the thickness of solid polymer electrolyte (SPE) decides the energy and power densities in SS-LMBs^36^. To verify this with respect to our best optimized cathode composition and morphology of LFP-5, we performed long-term cycle performance studies at higher current rate with different thickness of a polymer nanocomposite electrolyte containing 10 wt% TiO_2_ nanoparticles distributed in a PEO polymer matrix, referred to as SPNE^37^. The corresponding discharge capacity curves for three different thicknesses of SPNE (40, 90 and 130 µm) for the first 500 cycles at 2C at 70 °C are shown in Fig. 8a. It is interesting to note that with decreasing thickness of SPNE, the specific capacity and the cycle stability of the cell increase. This clearly indicates that the thickness of electrolyte has a huge influence on the electrochemical performance even with the standardized cathode. Even at 2C, the cells retained capacity values of 82 mAh g^-1^, 63 mAh g^-1^ and 28 mAh g^-1^ for the thickness of SPNE of 40 μm, 90 μm and 130 μm even after 500 cycles C with above 90% of coulombic efficiency, which indicates highest cycle stability of the cathode with the lowest SPNE thickness of 40 µm. In addition, we also studied the influence of temperature on the electrochemical behavior of the optimized LFP-5 cathode. Figure 8b shows the cycle performance of LFP-5 cathode measured at 0.2C at 30 °C and the corresponding charge–discharge curves are given in the in-set. Initially, the cells delivered high discharge capacity of 114 mAh g^-1^ and retained the capacity value of 96 mAh g^-1^ after 50 cycles C with good coulombic efficiency. The high discharge capacity of Li/SPNE/LFP-5 cell is mainly due to optimum electrode composition as well as high lithium salt content in the electrode which acts as plasticizer and increases the overall lithium ion conduction pathways within the electrode at low temperature (supported by DSC of EO/Li = 12; Figure S2). However, the Li/SPNE/LFP-5 cell at 30 °C maintain a large overpotential of 620 mV (in-set of Fig. 8b), which is mainly due to high crystallinity of SPNE at low temperature (30 °C). The LFP content and the electrochemical performance of this cathode might be further improved by using novel polymer electrolytes with amorphous structure and high lithium ion transport number and ionic conductivity as additives to cathode.

Though, the discharge capacity values delivered by the present LFP-5 cathode are comparable with the reported literature at low temperature (30 °C), superior discharge capacities are exhibited at high C rate in our case at 70 °C. In order to get an impression of the performance of our LFP-5 cathode in combination with some of the published data using polymer nanocomposite solid electrolytes and different LFP cathodes, Table 5 summarizes the reported discharge capacities at different temperatures and C-rates. The excellent electrochemical performance of LFP-5 cathode at high current rates is attributed to the densification of electrode to form non-porous and crack-free architecture, small particle size of LFP via ball milling, incorporation of conductive graphite as additive and optimum active material, lithium salt and binder content in the electrode. This cathode can also be used as universal electrode for all PEG-based solid electrolytes. Furthermore, this electrode composition and morphology of each cathode system needs to be studied and optimized for other high operating voltage cathodes as well in combination of suitable electrolytes instead of PEO systems to make the SS-LMB with high specific capacity and cycling stability feasible at room temperature.

**Table 5:** comparison of electrochemical performance of the present Li/SPNE/LFP-5 cell with those reported in the literature.

Cathode	Act. mater. content (%)	Electrolyte	Cell testing Temp. (°C)	Final capacity (mAh g^-1^)	C rate	Ref
**Electrochemical measurements above the melting temperature of PEO (> 50 °C)**						
LFP	70	PEO/PMHS/LiTFSI/SiO_2_	60	121 (50th cycle)	0.1	38
LFP	70	PEO/LiTFSI/Al_2_O_3_	60	136 (300th cycle)	0.2	39
LFP	60	PEO/LiClO_4_/SiO_2_	55	81 (90th cycle)	0.2	40
LFP	60	PEO/LiTFSI/PI	60	125 (30th cycle)	1	11
LFP	70	PEO/LiTFSI/TPU	80	119 (100th cycle)	1	41
LFP	70	PEO/LiTFSI/Mg_2_B_2_O_5_	50	120 (250th cycle)	1	15
LFP	80	PEO/PVDF/LiTFSI/Al_2_O_3_	50	~ 110 (30th cycle)	1	10
LFP	85	PEO/LiTFSI/Al_2_O_3_	105	100 (260th cycle)	1	42
LFP	70	PEO/LiTFSI/Ni_3_– (BTC)_2_	70	~ 74 (50th cycle)	1	2
LFP-5	63	PEO/LITFSI/P25	70	128(50th cycle)	1	This work
LFP-5	63	PEO/LITFSI/P25	70	82 (500th cycle)	2	This work
**Electrochemical measurements below the melting temperature of PEO (30 °C)**						
LFP	60	PEO/LiTFSI/PI	30	~ 100 (60th cycle)	0.5	11
LFP	80	PEO/PVDF/LiTFSI/Al_2_O_3_	30	98 (410th cycle)	0.1	42
LFP	70	PEO/LiTFSI/Mg_2_B_2_O_5_	30	50 (15th cycle)	0.2	15
LFMP	80	GPE/LLZO/GPE	30	~ 100 (200th cycle)	0.1	43
LFP	80	PEO/LLZTO	30	~ 104 (200th cycle)	0.1	44
LFP-5	63	PEO/LITFSI/P25	30	96 (50th cycle)	0.2	This work

## Conclusions

In this study we have investigated the influence of conductive additives (such as super C65 conductive carbon black and conductive graphite) as well as cathode composition on the electrochemical performance of LFP cathode for high performing SS-LMBs. The application of ball milling during the slurry preparation helps in decreasing the LFP particle size and also increase the homogeneity throughout the electrode. Further, the SEM images reveal the significance of calendaring process in densifying the electrode to form a non-porous and crack-free structures. Among all the cathodes, LFP-5 with weight ratio of LFP:CB:CG:PEO:LiTFSI = 63:4.9:2.1:19.4:10.6 delivered high discharge capacity of 141 mAh/g at 1C rate and retained 128 mAh g^-1^ upto 50th cycle at 70 °C. Thus, the present study reveals that the incorporation of 2wt% conductive graphite helps in increasing the electron conduction pathways more efficiently within the electrode and enhances the overall electrochemical performance of the cell. When the current rate was switched to 2C the Li/SPNE/LFP-5 cell delivered high discharge capacity 82 mAh g^-1^ even at 500th cycle at 70 °C. In addition, the cell also operates at low temperature of 30 °C and delivered a high initial discharge capacity of 114 mAh g^-1^ and retained 84% of its initial capacity at 50th cycle. The present results give the scope for the development of high performing SS-LMBs and the electrochemical performance of the LFP-5 cathode at low temperatures can be further enhanced by using novel amorphous solid electrolytes, additives and decreasing the solid electrolyte thickness. The future perspective for increasing the specific energy of the cell: (1) by increasing the active material content in the electrode using minimal amount of highly ion conductive medium (novel solid electrolytes), (2) increasing the thickness of electrode or (3) by using high operating voltage cathodes.

## Methods

### Materials

Standard LiFePO_4_ cathode, Super C65 conductive carbon black, conductive graphite and carbon-coated aluminium current collector was procured from MTI corporation. PEO (50,00,000 g/mol), bis(trifluoromethanesulfonyl)imide lithium salt (LiTFSI, 99.95%) and lithium metal (99.9%, 380 μm) are from Sigma Aldrich. Acetonitrile and n-methyl pyrrolidine are from Alfa Aesar and P25 (TiO_2_ nanoparticles) was purchased from Degussa.

### Preparation of cathode slurry

Initially, the cathode slurry was prepared according to the procedure reported in the literature^22^. Typically, the cathode composition of LFP, super 65 carbon, PEO and LiTFSI (EO/Li = 20:1) was maintained in the weight ratios of 63:7:22.7:7.3 and the resulting mixture was stirred overnight using acetonitrile as solvent (50 ml of acetonitrile is added for 1gm of PEO) to form an homogenous slurry. Then the resulting slurry was coated onto the carbon-coated aluminium current collector by using a doctor blade and then dried at room temperature for 4 h. After drying at room temperature, the electrode was vacuum dried in the oven at 50 °C for overnight and the dried electrode was densified by calendaring machine (heat roll press machine, Model-TMAX-JS100; distance between the rolls was about 30 μm and a constant roll temperature of 40 °C was maintained throughout the densification process). Finally, the electrodes were punched into the size of 14 mm diameter by using disc cutter and used for the fabrication cell. In the present study, we have also prepared different electrodes by reduced the solvent content to 75% (vol) as well as increased the lithium salt content (with different EO/Li ratios of 20:1, 16:1 and 12:1) during the cathode preparation to decrease the porosity in the electrode and to enhance the electrochemical performance.

Furthermore, ball milling method has been implemented in order to prepare the cathode slurry. For this, initially, 122.9 mg of LiTFSI is dissolved in 8 ml of Acetonitrile and stirred for 10 min to form a transparent solution. Then 226.9 mg of PEO was added slowly to the above solution and stir for overnight. Weigh separately 733 mg of LFP, 81.4 mg of super C65 carbon and transfer it into a zirconia ball mail vial (with a capacity of 25 ml) and the above mixture of PEO-LiTFSI solution is added to it, so that the complete composition of the cathode corresponds to the weight ratio of 63:7:19.4:10.6. Finally, 0.5 ml of NMP is added into the vial and then ball milled by using Retsch cryomill 200 for 2 h at room temperature with 10 min interval of resting time during each cycle. After milling, the cathode slurry was coated onto the C-coated aluminium current collector and follows the same procedure as mentioned above. Further, the cathode slurries with different weight percent of conductive graphite additive and with different electrode compositions (increasing the active material content) were also prepared by using ball milling process according to the electrode composition which are listed in Tables 3 and 4. The amount of active material loading on the electrodes are in the range of 1.2 to 2.6 mg cm^-2^.

### Preparation of PEO-LiTFSI-P25 electrolyte

Initially, 10 wt% of P25 (TiO_2_ nanoparticles) was dispersed in acetonitrile solution by using ultrasonication process for 1 h. Then, LiTFSI salt is added to the above solution followed by adding PEO to it and stirred for overnight in order to have better homogeneity of P25 particles in the polymer matrix. The EO/Li ratio is maintained as 20:1 according to the literature report^22^, due to its good mechanical stability and high ionic conductivity. Then, the solution is poured into the petri dish and vacuum dried in the oven at 60 °C overnight. The self-standing solid polymer nanocomposite electrolyte (SPNE) with a thickness of ~ 50 μm and 16 mm dia., is prepared by hot-press machine and used for assembling the cells. In addition, three other different thickness such as 40 μm, 90 μm and 130 μm was also prepared in the same way as mentioned above and used for comparative study at high C rate.

### Characterization

The surface morphology all the electrodes were analysed by Scanning Electron Microscopy (SEM; Zeiss LEO 1530). The particle size analysis of the SEM back scattered images was analysed by using Digital Surf, mountainsSEM Expert V9, particle analysis using threshold detection. Differential scanning calorimetry (DSC) measurements of PEO:LiTFSI with different EO/Li ratio of 12:1, 16:1 and 20:1 were performed on a Mettler Toledo DSC 3 at a heating rate of 10 K min^−1^ under nitrogen atmosphere.

### Electrochemical measurements

The electrochemical performance of the prepared LFP cathodes were evaluated by using lithium metal as reference electrode and SPNE as solid electrolyte. All the cells were fabricated in argon filled glove box. Prior to the measurement, the cells were stabilised at 70 °C for 12 h in order to have better contact between the electrodes and electrolyte. The galvanostatic charge–discharge and cycle performance measurements were conducted on BioLogic VMP3 potentiostat in the operating potential range of 2.5–4.2 V vs Li^+^/Li and all the capacity values were calculated based on the active material (LFP) content in the electrode.

## Supplementary Information

Supplementary Information 1.
