# Supplementary material for: Tuning of composition and morphology of LiFePO4 cathode for applications in all solid-state lithium metal batteries
Source: Sci Rep. 2022 Mar 31;12:5454. doi: 10.1038/s41598-022-09244-3 (PMC8971424; doi:10.1038/s41598-022-09244-3)
Supplement: Supplementary file 1 — Supplementary Information 1. [file 41598_2022_9244_MOESM1_ESM.file]

**Supporting Information**

**Tuning of Composition and Morphology of LiFePO_4_ Cathode for Applications in All Solid-State Lithium Metal Batteries**

**Erabhoina Harimohan^1,3^, Mukundan Thelakkat^1,2,3*^**

^1^Applied Functional Polymers, Universitätsstraße.30, University of Bayreuth, Bayreuth 95447, Germany

^2^Bavarian Polymer Institute, Universitätsstraße.30, University of Bayreuth, Bayreuth 95447, Germany

^3^Bavarian Centre for Battery Technology (BayBatt), Universitätsstraße.30, University of Bayreuth, Bayreuth 95447, Germany

Corresponding author E-mail: [Mukundan.Thelakkat@uni-bayreuth.de](mailto:Mukundan.Thelakkat@uni-bayreuth.de)


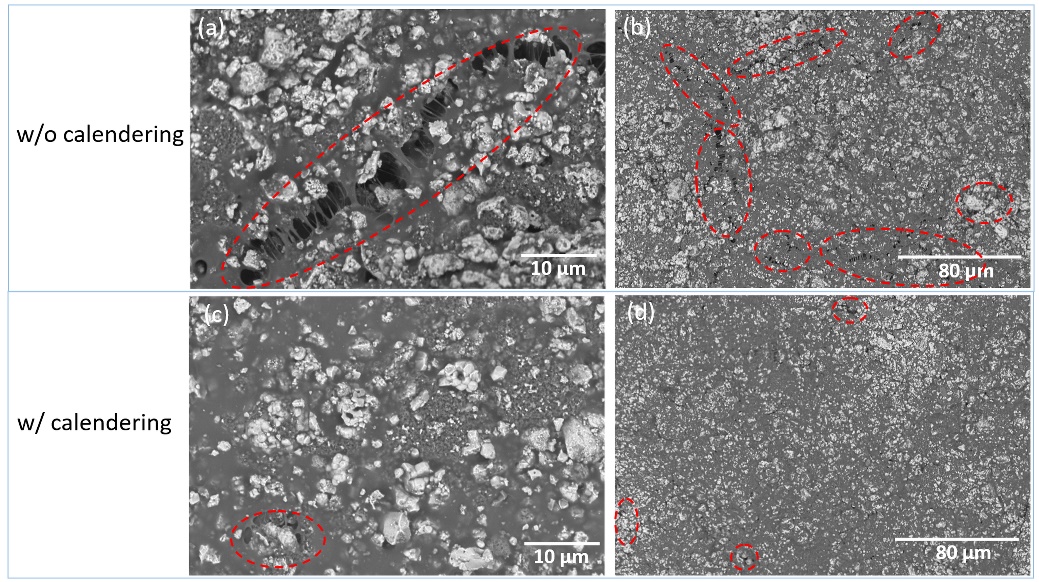


Figure S1. SEM images of LFP-1 (a) and (b) before, and (c) and (d) after calendaring process measured at different magnifications.


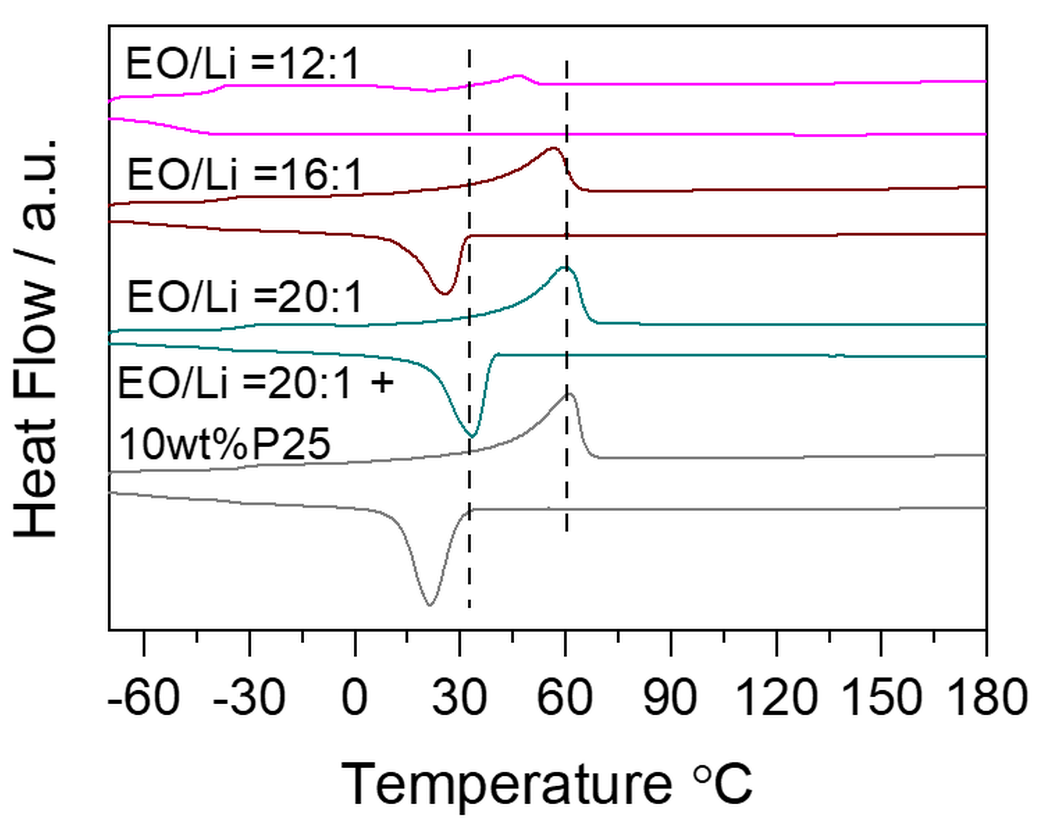


Figure S2. DSC curve of PEO and LiTFSI with different EO:Li ratios used as electrolyte additives in the cathode and SPNE.

Table S1. Summary of thermal properties of electrolyte additives used in the cathode and SPNE.

| Electrolyte additives | EO/Li = 12:1 | EO/Li = 16:1 | EO/Li = 20:1 | SPNE |
| --- | --- | --- | --- | --- |
| T_m_ /⁰C (ΔH_m_ / J.g ^−1^) | 4.1 | 49 | 57 | 62 |
| T_g_ /⁰C | -40 | -37 | -33 | -31 |


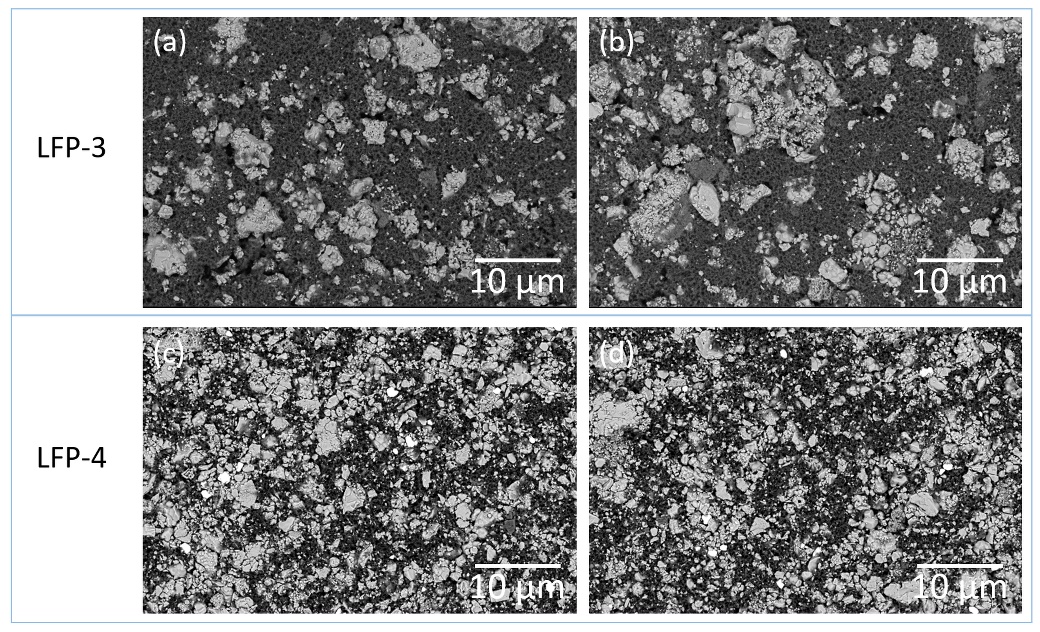


Figure S3. SEM back scattered images of (a) & (b) LFP-3 and (c) & (d) LFP-4 taken at different places on the electrodes.


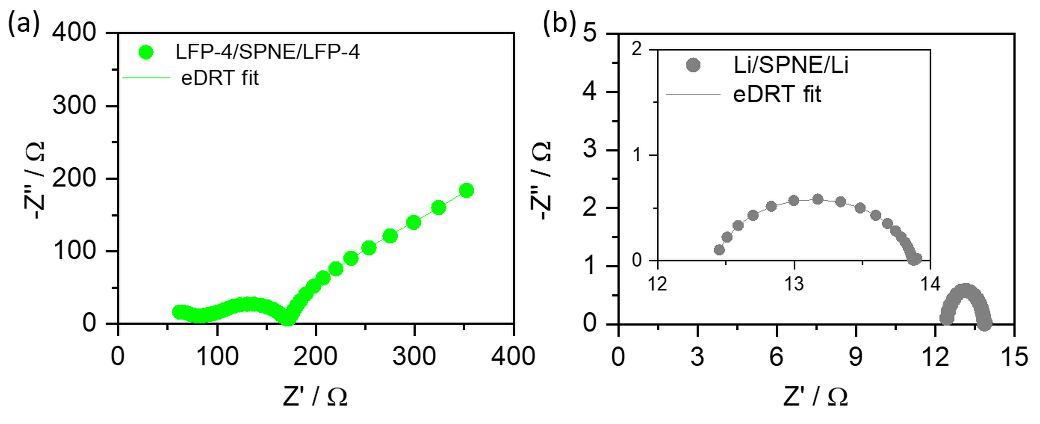


Figure S4. Nyquist plots and the corresponding eDRT fits of (a) LFP-4/SPNELFP-4 and (b) Li/SPNE/Li.


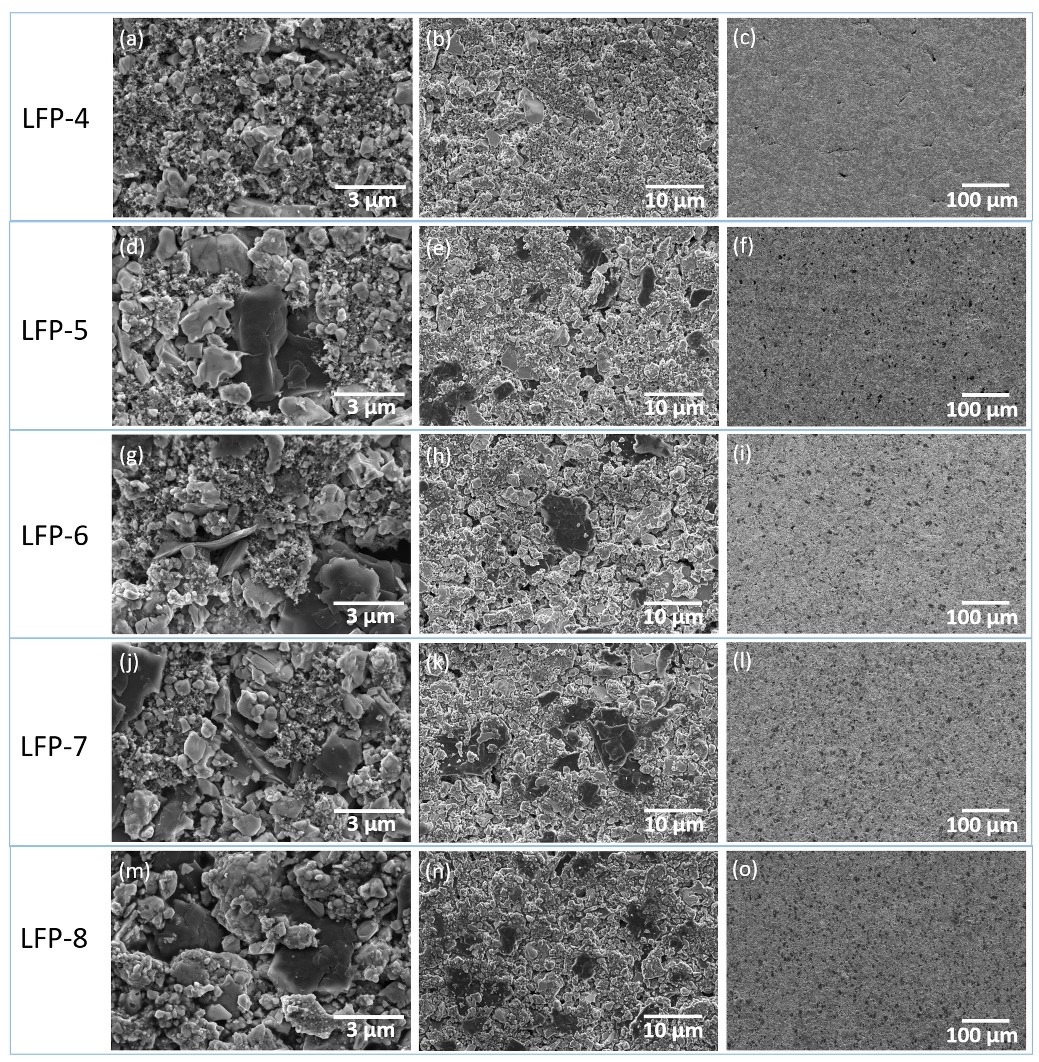


Figure S5. SEM images of calendered LFP-4, -5, -6, -7, -8 cathodes measured at different magnifications.


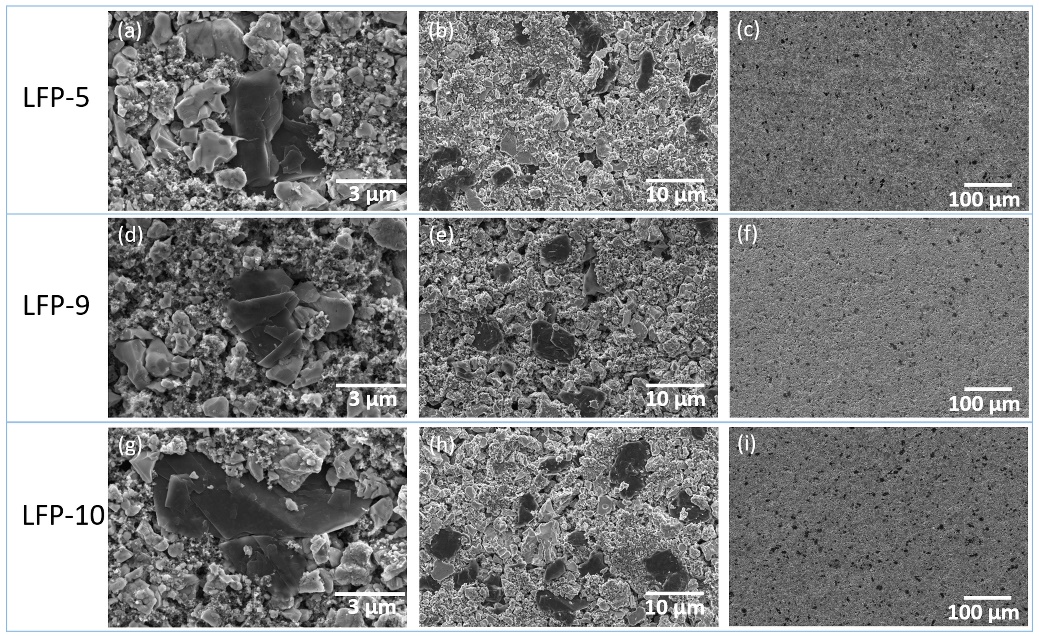


Figure S6. SEM images of calendered LFP-5, -9, -10 cathodes measured at different magnifications.


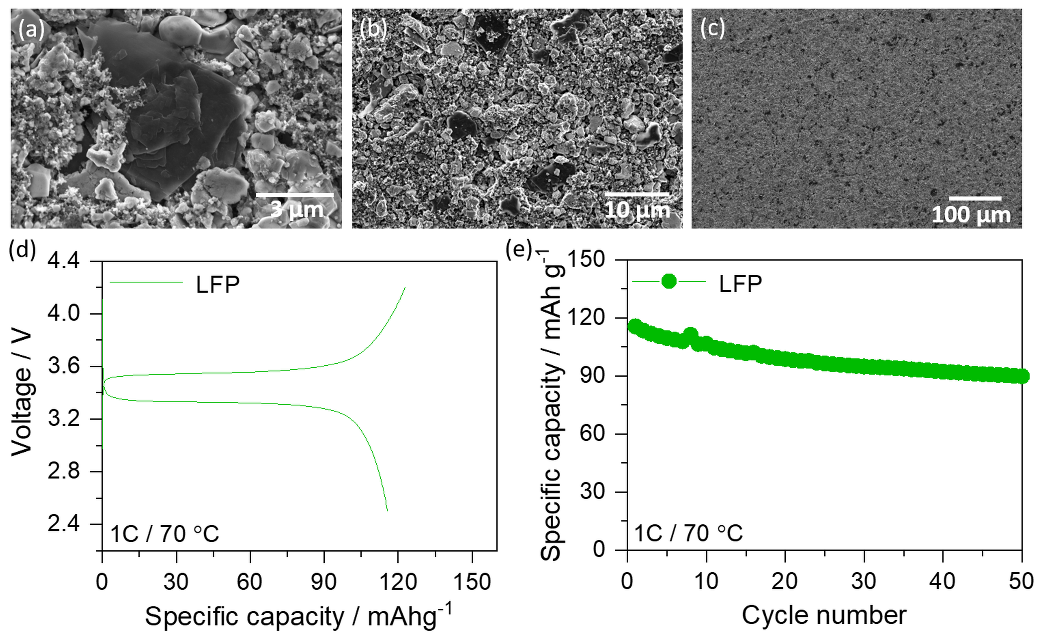


Figure S7. (a) to (c) SEM images of calendered cathode with 56 wt% LFP content measured at different magnifications, (d) and (e) are the charge/discharge and cycle performance curves of Li/SPNE/LFP (with 56 wt% LFP content) cell measured at 70 ᴼC at 1C rate.
